# Non-protein coding RNA sequences mediate specific colorimetric detection of *Staphylococcus aureus* on unmodified gold nanoparticles

**DOI:** 10.1038/s41598-022-16551-2

**Published:** 2022-07-23

**Authors:** Subash C. B. Gopinath, Santheraleka Ramanathan, Suresh V. Chinni, Vicneswarry Dorairaj, Thangavel Lakshmipriya

**Affiliations:** 1 Institute of Nano Electronic Engineering, Universiti Malaysia Perlis, 01000 Kangar, Perlis Malaysia; 2 Faculty of Chemical Engineering Technology, Universiti Malaysia Perlis, 02600 Arau, Perlis Malaysia; 3 Department of Biochemistry, Faculty of Medicine, Bioscience, and Nursing, MAHSA University, 42610 Jenjarom, Selangor Malaysia; 4 Department of Biotechnology, Faculty of Applied Sciences, AIMST University, Bedong, Malaysia

**Keywords:** Biochemistry, Biomarkers, Nanoscience and technology

## Abstract

Nonprotein coding RNA (npcRNA) is a transcribed gene sequence that is not able to translate into protein, yet it executes a specific function in modulation and regulation mechanisms. As npcRNA is highly resistant to the mutation, the *Sau-02* npcRNA gene and its probe oligonucleotide, which are specifically present in *Staphylococcus aureus* and in methicillin-resistant *S. aureus* only, used to develop a highly specific and sensitive colorimetric assay on unmodified gold nanoparticles (AuNPs). Hybridization between the npcRNA *Sau-02* gene sequences was detected through noncrosslinking AuNP aggregation in salt solution in the presence of probe-target gene sequences. AuNPs of 10 and 15 nm in sizes with monovalent ion salt (NaCl) solution were optimized as the ideal tool for investigating the stability of AuNPs upon the addition of gene sequences. The state dispersed and aggregated forms of 10 nm AuNPs with the presented colorimetric assay were justified through field emission scanning electron microscopy and atomic force microscopy. The particle distribution of two different AuNP states was evaluated through particle distribution analysis. The lowest detection amount of *S. aureus* npcRNA from the colorimetric assay performed was 6 pg/µL, as the color of AuNPs turned blue with the presence of probe oligonucleotides and target gene sequences.

## Introduction

*Staphylococcus aureus* is a gram-positive bacterium known for its high resistance and adaptation to harsh conditions and the environment, including the development of resistance against numerous antibiotics commonly used to treat infections. A particular strain of this bacterial species, methicillin-resistant *S. aureus* (MRSA), is classified as a pathogenic organism by the WHO, as it confers resistance toward methicillin antibiotics, and infection by this bacterium leads to fatality^1^. MRSA infections are transmitted or acquired, particularly in nosocomial settings. Early and accurate detection of this pathogenic microorganism will help to intervene in pathogenesis. Thus, an appropriate method for the accurate detection of this pathogen would be of at most clinical advantage. Therefore, the current research is focusing on standardizing and developing a rapid method to identify *S. aureus* by applying principles of biosensors. Electrochemical immunoassays, and surface-enhanced Raman spectroscopy (SERS) sensing are the common types of strategy used in detecting *S. aureus* with the selective and sensitive ribonucleic acids (RNA) based detection. However, the recent studies have geared their efforts towards the development of rapid and sensitive strategies in a simple and straightforward technique. In addition, various nanoparticle-based biosensors are developed, where the output signal is determined through fluorescence, electrochemistry and many more advancements with the state-of-art microelectromechanical systems^2,3^. Yet, the intrinsic drawbacks in each system broaden the scope of detecting the sensitive single or double stranded RNA and deoxyribonucleic acid (DNA) with multiple conjugation of nanomaterials and sensing mechanisms^4,5^.

Nonprotein coding ribonucleic acids (npcRNAs) are RNA transcripts that do not code for protein synthesis directly but play numerous roles. However, npcRNAs, with ~ 500 nucleotides, are significantly involved in viral gene expression, pathogenic gene expression and several other biological expressions. Thus, npcRNAs have paved the way for developing a robust diagnosis^6–10^. Moreover, npcRNA-based oligonucleotide probes are highly specific only to *S. aureus* and MRSA with the *Sau-02* gene. npcRNA gene-based detection presents a comparatively specific and promising outcome as a convenient method, as npcRNA is sensitive to mutation^11,12^. Nonsynonymous point mutations in coding genes often lead to misleading identification of bacterial genes, while npcRNA mutations simply eliminate the need for diagnosis, as the functional properties of npcRNAs and the associated regulatory mechanism of cells are deactivated^10,13^.

The development of biosensors is a growing interdisciplinary field of nanotechnology. The development of colorimetric assays for detecting the presence of analytes of interest, even in complex samples, is one of the preliminary sensing methods undergoing significant amelioration^14,15^. Rapid detection and visible color changes to be detected through the bare eye qualitatively without the need for sophisticated equipment, add advantages to this assay in addition to the fact that this assay does not require or utilize any organic solvents, heavy metals, light-sensitive dye molecules or tagging steps, which can interfere with the detection steps^16–18^. Colorimetric assays are well established nanoparticle based strategy for detecting pathogens, which are equally rapid and sensitive to other immunoassays^19^. Gold nanoparticles (AuNPs) based colorimetric assays have become a high priority in research over the last decade, and they've been used to monitor (bio)chemical compounds^20^. AuNPs are widely used in colorimetric detection with the advantages of gold in nanometer dimensions, changing significantly in almost every aspect from their bulk properties. These include changes in its color, which due to the photoelectronic properties of the nanoparticles, define the intrinsic mode, which is directly related to its size, shape, composition, dielectric surroundings, electron confinement and surface chemistry or aggregation state, which is also known as the interparticle distance^21–23^. This unique photoelectronic property of AuNPs results from localized surface plasmon resonance (LSPR) phenomena that occur when electromagnetic radiation is incident on the particles. With increasing interest in basic and applied nanoscience, the use of metal nanoparticles, especially AuNPs, spans various fields, such as therapeutic purposes, biological sensors, and data storage, as well as in colorimetric assays for specific biomolecule detection^24^. A recent study conducted by Liu et al., cysteamine (CS)-stabilized gold nanoparticles were functionalized with *S. aureus*-specific pVIII fusion protein. The state of aggregation was determined by the visual color change in the presence of target *S. aureus*. The study proved the colorimetric detection of *S. aureus* with a low concentration of 19 CFU mL^−1^. The study has proposed the sensitive AuNPs based colorimetric assays for on-site detection of pathogenic bacteria^25^.

The advantages of AuNPs are well integrated in colorimetric assays to determine the presence of target gene sequences based on charge interactions. The principle behind genome-based colorimetric assays is that double- and single-stranded oligonucleotides (dsDNA and ssDNA) have different electrostatic properties^26^. The essential difference arises because ssDNA can uncoil sufficiently to expose its bases, whereas dsDNA has a stable double-helix geometry that always presents the negatively charged phosphate backbone. Repulsion between the charged phosphate backbone of dsDNA and the adsorbed citrate ions on AuNP solution dominates the electrostatic interaction between the nanoparticles and dsDNA, so that dsDNA will not adsorb. Since ssDNA is sufficiently flexible to partially uncoil its bases, it can be exposed to AuNPs^15,27^. Under these conditions, the negative charge on the backbone is sufficiently distant so that attractive van der Waals forces between the bases and AuNPs are sufficient to cause ssDNA to stick to the gold. The same mechanism is not applicable to dsDNA because the duplex structure does not permit the uncoiling needed to expose the bases^28,29^. With the above strategy, a npcRNA-based colorimetric assay is presented in this research. The npcRNA *Sau-02* gene was identified in gram-positive *S. aureus*. Probe oligonucleotides specific to the npcRNA *Sau-02* gene were designed as the target for the colorimetric assay. Complementary genome base pairing indicates the presence of pathogenic *S. aureus* bacteria. The electrostatic property of AuNPs is well optimized for detecting pathogenic bacterial strains through npcRNA-based colorimetric assays.

## Experimental procedure

### Materials and reagents

The bacterial isolates used in this study were acquired from AIMST University, Malaysia. All bacterial isolates were maintained at − 80 °C in the recommended storage solution and revived by inoculation into Luria broth (LB) media at 37 °C prior to usage. The prepared nucleic acids from the chosen bacterial strain *Staphylococcus aureus* were stored and preserved at − 20 °C until use. Unmodified AuNPs with 10 nm and 15 nm nanosizes at 0.1 mg/ml (0.01% V/W based on gold) were purchased from NANOCS, USA. Sodium chloride (NaCl, HmbG-2134230), potassium chloride (KCl, Sigma–Aldrich P9333-1 kg, batch: 059K0090), calcium chloride (CaCl_2_, Bendosen-C0196-2081701), and magnesium chloride (MgCl_2_, Sigma–Aldrich) were used as salt solutions for the colorimetric assay. An extraction kit to extract *S. aureus* genomes was procured from Invitrogen (Thermo Fisher Scientific, USA). The *Sau-02* gene probe oligonucleotide, as the specific target for *S. aureus* was obtained from NANOCS, USA.

### AuNP and salt solution optimization

Monovalent and divalent salt solutions were analyzed for AuNP aggregation as the primary optimization step for the colorimetric assay. Approximately 5 M stock solutions were prepared for each type of salt solution at room temperature. The set of salt solution concentrations was defined from 0.05 to 500 mM, with a series of tenfold dilutions from the stock solution. The salt solution was tested with both 10 nm and 15 nm AuNPs to investigate the degree of nanoparticle aggregation with respect to the size of the nanoparticles and the strength of the interparticles, depending on the monovalent or divalent ion solution. The colorimetric assay between salt solution and AuNPs was performed in 0.2 mL sterile Eppendorf PCR tubes. Approximately 3 µL of 10 nm and 15 nm AuNP solutions were added to PCR tubes. Then, 1 µL of prepared salt solution [0.05, 0.5, 5, 50, 500 (mM)] was added to the PCR tubes. Equal volumes of sterile distilled water were added to replace the biomolecules in a tube containing AuNPs, which acted as the control set for each assay. The color change took place in all the tubes within 10 min of reaction time. The reaction solution was then analyzed with spectrophotometry to examine and elucidate the AuNP aggregation with graphical presentation. These steps were repeated with every type of salt solution, and the results were evaluated to screen the optimum AuNP size, concentration, and type of salt solution for *S. aureus* genome detection using npcRNA through colorimetric assay.

### Staphylococcus aureus extraction

Nucleic acid of *S. aureus* was extracted using the Purification Kit (Invitrogen, Thermo Fisher Scientific, USA) according to the manufacturer’s instructions. Concisely, lysate was obtained by resuspending prepared cell pellets of *S. aureus* culture in a mixture of 800 µL lysis buffer and 100 µL lysis enhancer. Following incubation at 65 °C for 10 min, the mixture was homogenized by stirring for 10 min at maximum speed. Then, it was subjected to centrifugation at 14,000*g* at 4 °C for 2 min. The resulting aqueous phase was transferred into a fresh tube containing 900 µL of binding buffer per 500 µL of supernatant. The analytes were slightly vortexed. Then, the sample was centrifuged at 14,000 *g* at 4 °C for 1 min in a spin column-tube assembly (700 µL of sample per tube). The obtained pellet was washed with 500 µL of wash buffer and eluted with 100 µL of elution buffer. The extracted sample was stored at − 20 °C prior to use. The extracted sample was analyzed on a 1% agarose gel prepared by heat dissolving 0.5 g of molecular grade agarose powder in 50 mL of 1X TAE buffer. The warm gel was stained with a few (2–3) drops of ethidium bromide (EtBr) before being poured into the gel casting tray of the horizontal mini-subcell GT system (Bio–Rad Laboratories, USA) harboring an appropriate comb. After 20 min at room temperature, the solidified gel with the tray was submerged in 1X TAE buffer in the electrophoresis system. Then, the gel was run at 110 V for 45 min followed by visualization under UV imaging by placing the gel on a UV transilluminator connected to a gel image capture system (GelDocXR, Bio–Rad Laboratories, USA).

### Detection of npcRNA with AuNP colorimetric assay

Prior to use in the colorimetric test, the npcRNA of *S. aureus* was prepared in a set of 10 dilutions from a stock of 84 pg/µL and then snap cooled after 10 s of heat treatment at 95 °C by immediate freezing on ice until use. The prepared samples were sequentially added to a mixture of 1 µL of probe oligonucleotide and 3.5 µL of AuNPs. Subsequently, 0.5 µL of 6 M NaCl was added to the ensemble solution after an incubation period of not more than 5 min. The colorimetric assay for *S. aureus* npcRNA detection was evaluated with the color change and spectrophotometry analysis.

## Results and discussion

**Figure 1:**
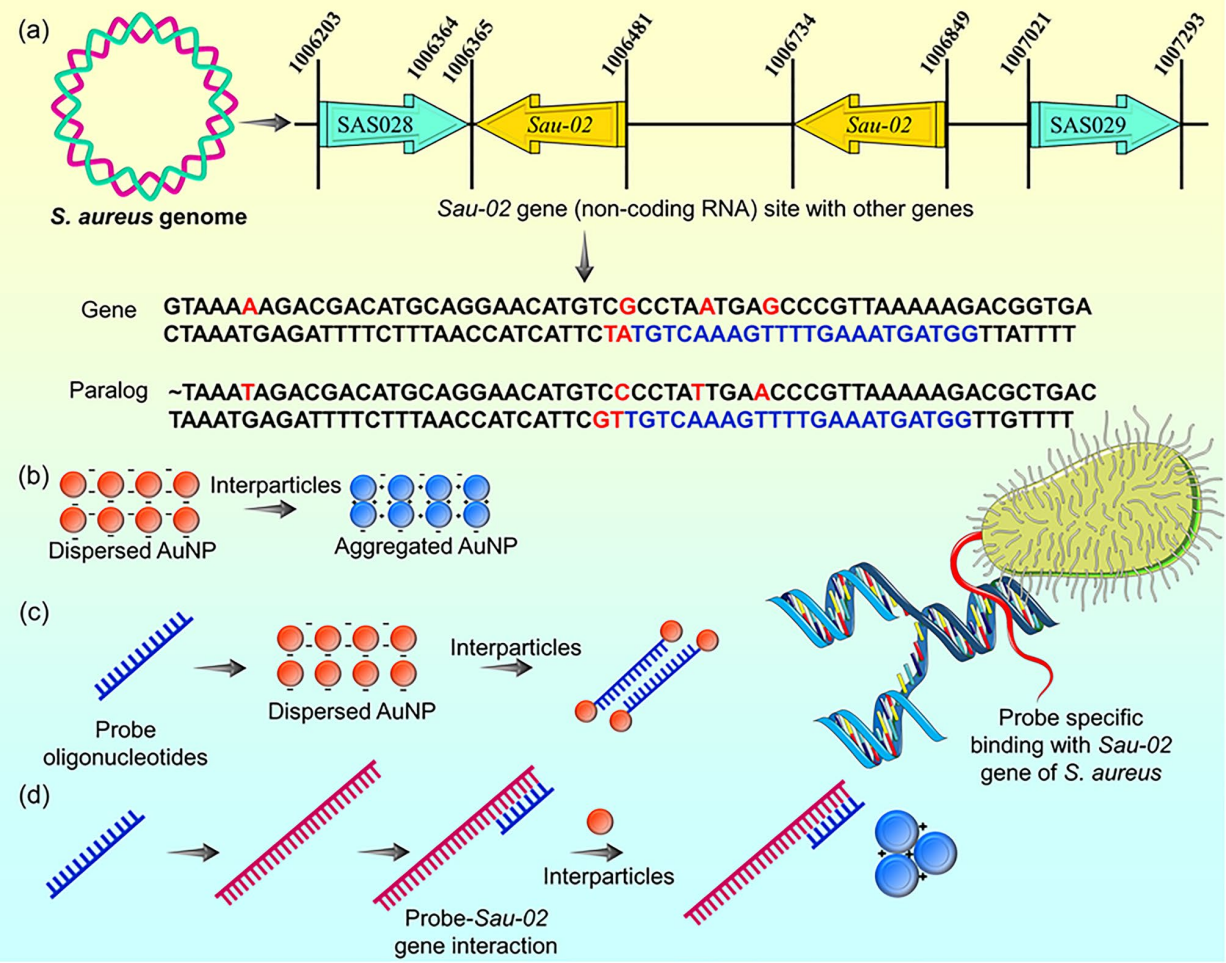
Schematic illustration of colorimetric assay strategies developed to detect pathogenic gram-positive *S. aureus* bacteria. (**a**) Illustration of the *S. aureus* genome and its ncpRNA *Sau-02* gene site with other genes. The genome sequence of the target is shown with gene and paralog sequences. The nucleotides colored blue in the sequence were applied as probe oligonucleotides for the colorimetric assay. (**b**) Colorimetric assay of AuNP with salt solution. AuNPs aggregated with salt interparticles, and the color of the solution changed from red to blue. (**c**) AuNP colorimetric assay incorporated with probe oligonucleotides. AuNPs remain red because of their high electrostatic affinity with probe oligonucleotides. (**d**) Strategy of detecting ncpRNA with the *Sau-02* gene using probe oligonucleotides through colorimetric assay.

The research presented a straightforward npcRNA-based colorimetric assay as the preliminary validation strategy to recognize the pathogenic gram-positive *S. aureus* bacterium. The augmentation between the electrostatic property of AuNPs and specific nucleic acid structures was well presented in the colorimetric assay, as shown in Fig. 1. The stability of AuNPs in monovalent and divalent ion-based salts was evaluated. Pathogenic *S. aureus* was detected based on its npcRNA gene sequence with AuNP stability in salt solution. The designed probe oligonucleotides recognized the specific npcRNA *Sau-02 gene*, which acts as the target for the npcRNA-based colorimetric assay.

### Gold aggregation colorimetric assay

#### Monovalent ion salt induced gold aggregation

**Figure 2:**
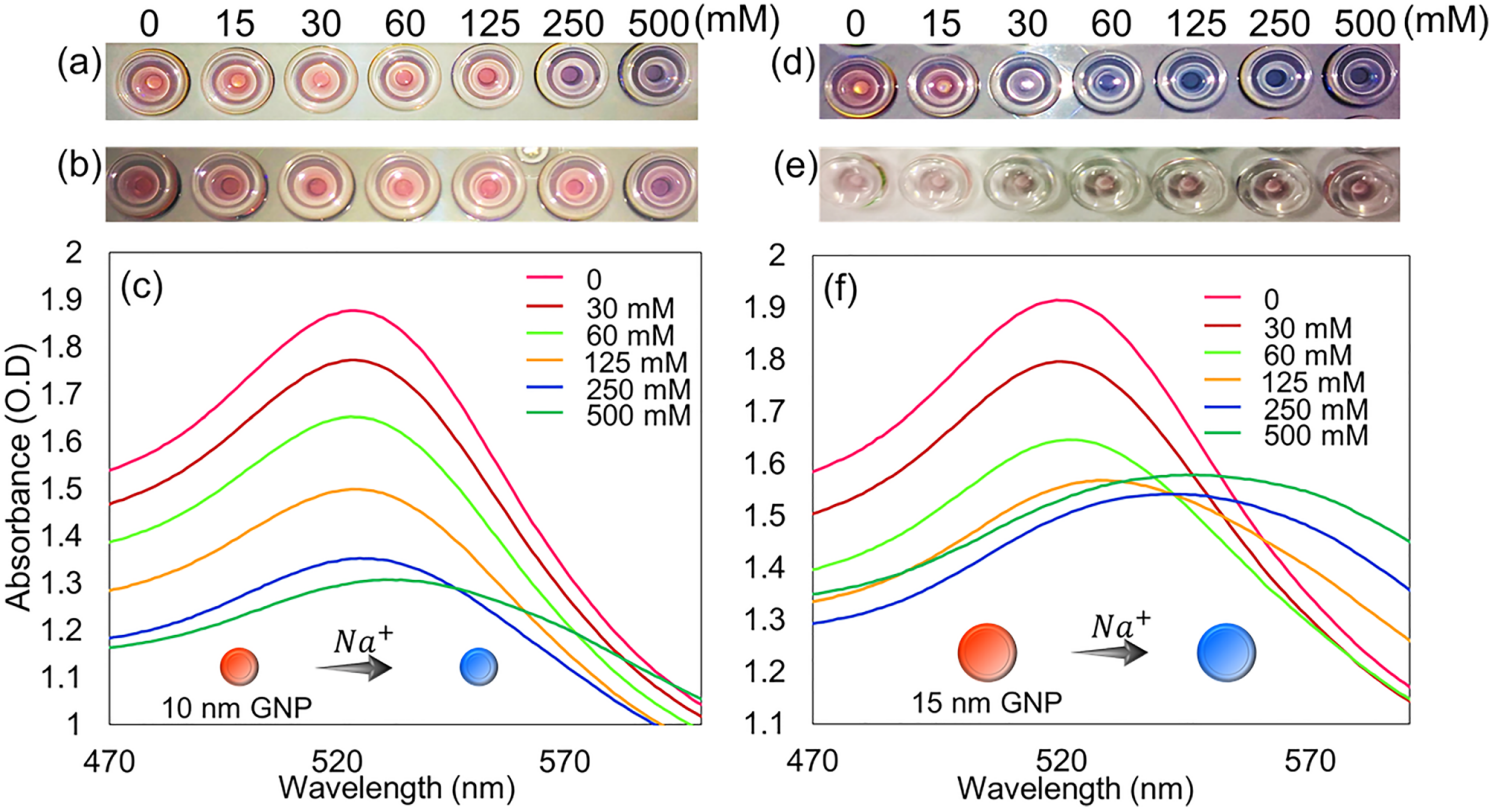
Colorimetry of AuNPs with monovalent salt solutions. Digital image of colorimetric assay performed with 10 nm AuNPs against (**a**) KCl solution and (**b**) NaCl solution. (**c**) Graph showing the peak position at 520 nm in the plasma spectrum, indicating the stability of 10 nm AuNPs at different NaCl concentrations. Digital image of colorimetric assay performed with 15 nm AuNPs against (**d**) KCl solution and (**e**) NaCl solution. (**f**) Graph illustrating the effect of larger AuNPs in sustaining stability against different NaCl concentrations.

The stability of AuNPs with monovalent ion salts was examined using NaCl and KCl salt solutions. The colorimetric assay was performed and analyzed with 10 and 15 nm AuNPs to examine the influence of nanoparticle size in sustaining its stability against monovalent ion salt solutions. Figure 2 shows the visual observation of AuNP dispersion and aggregation conditions based on the color of the colorimetric assay. Figure 2a,b show digital images of the colorimetric assay using KCl and NaCl monovalent salt solutions with 10 nm AuNPs, respectively. The AuNP color remained red in both sets when no salt solution was added. It reveals the dispersed state of AuNP. As the monovalent ion salt was added, the color of AuNPs remained red from 15 to 125 mM KCl and changed to purplish blue at 250 mM and 500 mM (Fig. 2a). This reveals that 10 nm AuNPs are stable until 125 mM KCl and lose stability at 250 mM, as AuNP aggregation is denoted by the purple AuNP solution. Figure 2b shows that 10 nm AuNPs are stable up to 250 mM NaCl and aggregate with 500 mM monovalent ion salt, which was reflected by the purple color of the AuNP solution observed. Figure 2c shows the absorbance reading examined for 10 nm AuNPs in monovalent NaCl salt solution. The unique properties of AuNPs associated with localized surface plasmon resonance (LSPR) phenomena were well explained by the optical extinction of AuNPs and the collective oscillation of free conductive electrons known as plasmon resonance, also known as quantum mechanical quasiparticles. The peak observed at 520 nm indicates the stability of AuNPs. The peak at the control set shows dispersed and stable AuNPs. The height of the peak at 520 nm decreased as the concentration of NaCl solution was added to the AuNPs. This indicates that the AuNPs aggregate and lose their stability. Thus, the peak shifts from blue (520 nm) to red light in the plasma spectrum^30^. Based on the absorbance reading, 10 nm AuNPs totally aggregated with 500 mM NaCl solution as the plasma curve flattened at 520 nm and shifted to the right in the spectra. Figure 2d shows the color change that occurred with the colorimetric assay performed between KCl solution and 15 nm AuNPs. The digital image indicates that 15 nm AuNPs are atypical against monovalent KCl salt, as the color change observed in the lowest KCl solution (15 mM). The plasma spectrum of 15 nm AuNP stability against KCl is shown in Fig. S1. The stability of 15 nm AuNPs against NaCl solution is shown in Fig. 2e and denotes the color change from red to purple. The colorimetric assay was further analyzed through the absorbance reading shown in Fig. 2f. The lower peak position observed for the high-concentration NaCl solution justifies the aggregation of AuNPs.

#### Divalent ion salt induced gold aggregation

**Figure 3:**
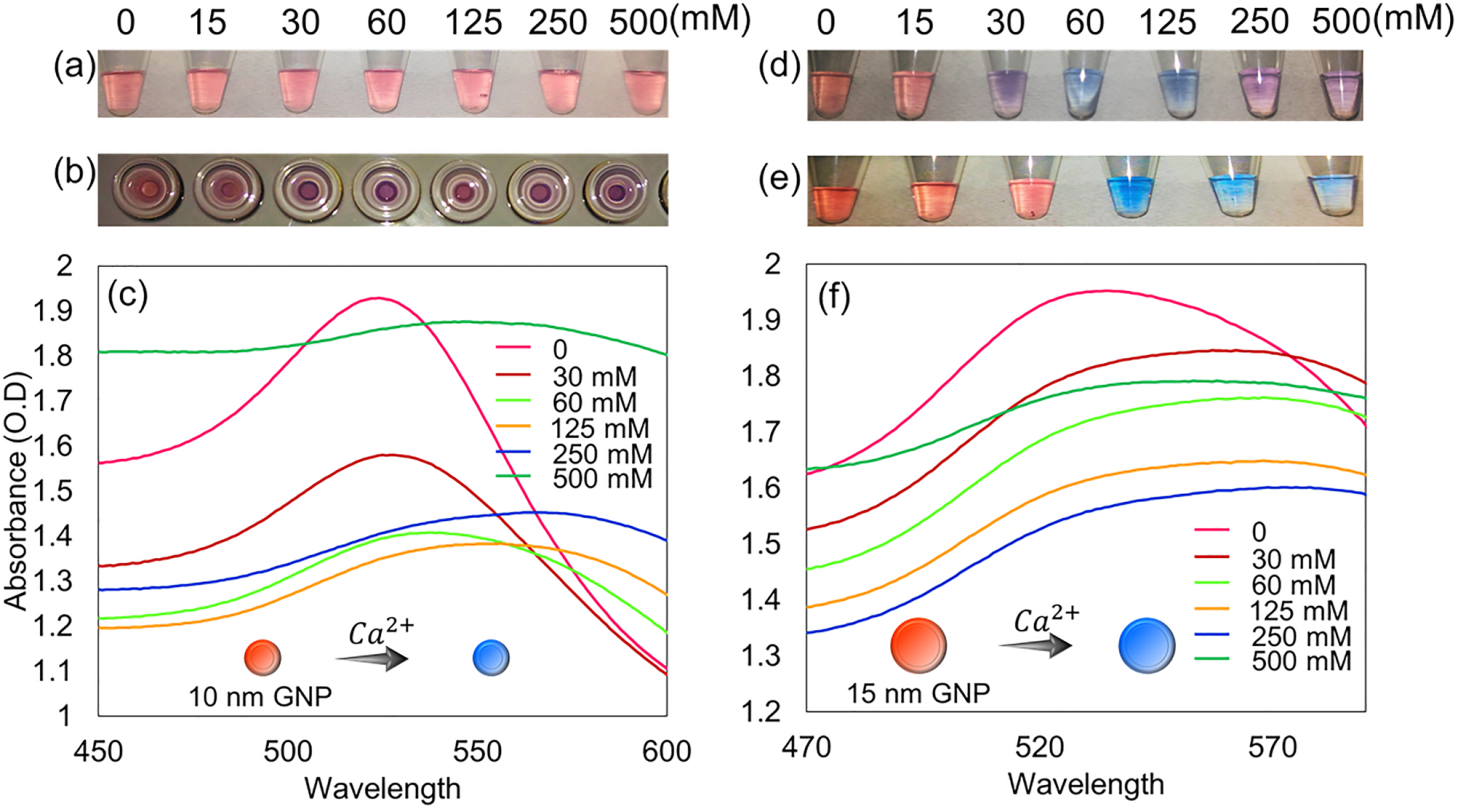
Colorimetry of AuNPs with divalent salt solutions. Digital image of colorimetric assay performed with 10 nm AuNPs against (**a**) MgCl_2_ solution and (**b**) CaCl_2_ solution. (**c**) Graph showing the scattered curve trend in the plasma spectrum, indicating the stability of 10 nm AuNPs at different CaCl_2_ concentrations. Digital image of colorimetric assay performed with 15 nm AuNPs against (**d**) MgCl_2_ solution and (**e**) CaCl_2_ solution. (**f**) Graph illustrating the effect of Ca^2+^ divalent ions as salt solutions in aggregating 15 AuNPs, shown by peaks in the 520 nm plasma spectrum.

The influence of divalent ion salt solution in disrupting the stability of AuNPs was evaluated using MgCl_2_ and CaCl_2_ solutions. Figure 3a shows the tubes of 10 nm AuNPs mixed with MgCl_2_ solution. The digital image shows that the color of AuNPs remains red at all concentrations of MgCl_2_, indicating that the divalent ion salt is not strong enough to disturb the stability of AuNPs. The AuNP stability against divalent ion salt was further examined using CaCl_2_. The digital image shown in Fig. 3b indicates that 10 nm AuNPs aggregated at a 30 mM salt concentration as the color of the solution changed from red to purple. This result is in good agreement with the corresponding plasma peak observed at 520 nm, shown in Fig. 3c. The peak position decreases drastically at 30 mM from the control and continues to shift to the red light of the plasmon spectrum. Moreover, the divalent ion salt strength was examined with 15 nm AuNPs. Figure 3d,e shows digital images of the colorimetric assay performed using MgCl_2_ and CaCl_2_ solutions, respectively. The images show that 15 nm AuNPs lose stability at salt concentrations of 30 mM and 60 mM, and an obvious AuNP blue color is visible. The aggregated AuNP color differentiates between blue and purple in Fig. 3d. The color variation may cause by several factors. The AuNP aggregation with monovalent and divalent ion-based salt are influenced by the acid dissociation constant (pK_a_)of the charged ion. The pK_a_ of charged ion directly impacts on the degree of AuNP aggregation, excluding the concentration and volume of salt added to the nanoparticle. The variation in AuNP aggregation state is visually determined by the color variation in aggregated AuNP solution^31^. Hence, the difference in the blue and purple color of aggregated AuNP in Fig. 3d may occurred to variation in the pK_a_ of divalent ion at different concentrations^32^. The plasma spectrum of 15 nm AuNP stability against MgCl_2_ is shown in Fig. S1. The absorbance reading recorded for the colorimetric assay with 15 nm AuNPs is shown in Fig. 3f. The aggregation of 15 nm AuNPs against the divalent ion salt is justified through the lower peak positions observed in the plasma spectra.

#### Optimized condition for gold colorimetric assay

AuNPs in the presence of salt interparticles can be readily understood by electrical double layer (EDL) theory. The use of balanced interparticles (salts/ions) exerts attractive or repulsive forces to cause aggregations or dispersions, respectively, which are due to the gain or loss of surface charges and predominantly rely upon the distance-dependent optical properties of AuNPs. The presence of positively charged ions (interparticles) destabilizes colloidal AuNPs, thus irreversibly instigating aggregates. On the other hand, the dispersed and negatively charged unmodified AuNPs uphold a strong absorbance in the 520 nm UV plasmon spectrum, which instantly aggregates with positively charged interparticles and shifts to the red light of the plasmon spectrum. The result of colorimetric assay is evaluated through bare eye investigation by analyzing the red or blue color solution^33,34^. The colorimetric assay works with the concept of chemistry as explained above to detect different biomolecules. However, the optimized conditions of AuNPs and salt solution are compulsory to evaluate for each type of colorimetric assay, depending on the type of biomolecules targeted to recognize in the assay. In the present research, the size of AuNPs and the strength of salt were optimized upon AuNP stability to detect the npcRNA gene of *S. aureus*. Based on Figs. 2 and 3, 10 nm AuNPs are more stable than 15 nm AuNPs. The color changes were quicker with 15 nm AuNPs with both monovalent and divalent salt solutions than with 10 nm AuNPs. As the size of AuNPs increases, the plasmonic coupling strength increases. Thus, the rate of aggregation is rapid with positively charged interparticles, which enables the quick color change of AuNPs. Under some conditions, large AuNPs tend to undergo self-aggregation, depending on their external domain. Thus, 10 nm AuNPs were chosen as the ideal size for the colorimetric assay designed in the present research. The colorimetric assay strategy was investigated for the influence of monovalent and divalent ion salt solutions. The results obtained from the colorimetric assays indicate that both monovalent and divalent ions have similar effects in inducing aggregation with AuNPs. The insight examination of surface plasmon spectra reveals that the NaCl-based assays showed a stable spectral shift from the lowest to the highest concentration of the salt. However, the changes were scattered with divalent ions, although the aggregation justified. CaCl_2_ and MgCl_2_ may be involved in AuNP ligand interactions. Moreover, calcium and magnesium have higher affinity toward the nucleic acid base phosphate group and may result in several disruptions in the colorimetric assay involving genomic sequences. Hence, NaCl salt solution was selected to be used to induce positive interparticles for the colorimetric assays.

#### Boost gold stability with probe oligonucleotide

**Figure 4:**
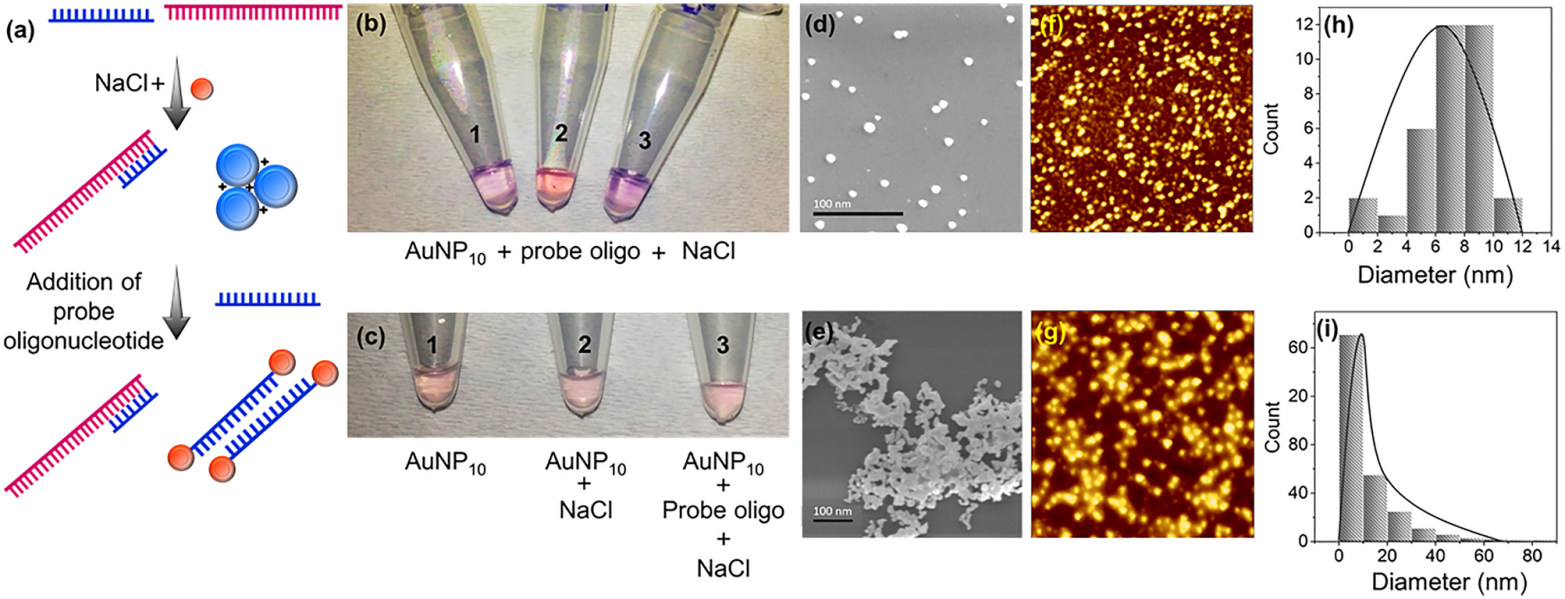
(**a**) Schematic illustration of the colorimetric assay performed with the *Sau-02* npcRNA gene and probe oligonucleotides. The addition of probe oligonucleotides changes the color of AuNPs from blue to red. (**b**) Triplicate colorimetric assay experiments using probe oligonucleotides. (**c**) Gradual addition of analyte in colorimetric assay (Tube 1–3). FESEM image of (**d**) dispersed and (**e**) aggregated 10 nm AuNPs. (**f**) Dispersed and (**g**) aggregated AuNPs viewed by optical AFM imaging. Particle size analysis of AuNP. (**h**) Normal distribution histogram showing dispersed AuNPs. (**i**) Skewed right histogram showing the nonnormal distribution of aggregated AuNPs.

Colloidal 10 nm AuNPs with 500 mM NaCl solution were used to perform a colorimetric assay to detect npcRNA of *S. aureus* using probe oligonucleotides. Figure 4a shows a schematic illustration of the colorimetric assay strategy in detecting the npcRNA of pathogenic bacteria. The specific binding of probe oligonucleotides with the npcRNA sequence (complement sequence) attains a highly stable double helix structure, which does not have any interaction with AuNPs. Thus, AuNP aggregates and the visible light color change to blue in the presence of NaCl solution. AuNP stability was examined by adding more probe oligonucleotides into the colorimetric assay. As the specific npcRNA genomic sites for the probe are occupied by prehybridization, the free single-stranded probes attracted toward the AuNPs. Due to the high electrostatic affinity of AuNPs, aggregation occurs anonymously, and the color changes to red. Figure 4b shows the assay performed to investigate the electrostatic affinity between single-stranded probe oligonucleotides and AuNPs. The assay performed with triplicate repetition revealed that the color of tube 2 remained red, whereas the other tubes changed to purple. There are two possibilities for the false positive result of tubes 1 and 3. The amount of probe oligonucleotide added into the assay may not be sufficient to inhibit AuNP aggregation with salt solution. Since the sensitivity of AuNPs is prominent, the simplest errors, such as handling and analytical measurements, may result in false positive results. Figure 4c shows the tubes of AuNPs with the steps of investigating the AuNP stability in the presence of probe oligonucleotides. Tube 1 shows the red color of AuNPs, and it was changed to purple (Tube 2) upon the addition of NaCl solution. Then, probe oligonucleotides were added to identify the AuNP stability (Tube 3). The purple color has faded. This may result from upgraded AuNP stability in the presence of excess single-stranded probe. However, the monovalent salt solution is strong and does not release AuNPs easily, although the electrostatic affinity of AuNPs toward the probe is high. Therefore, it is mandatory to add salt solution as the final analyte to obtain an accurate colorimetric outcome.

#### Insight of AuNP stability

The stability of AuNPs in colorimetric assays was further understood with electron and optical microscopic characterization to visualize the scattered and aggregated AuNPs with and without the presence of salt solution. Figure 4d shows scattered AuNPs 10 nm in size observed under a field emission scanning electron microscope (FESEM, Hitachi, S-4300 SE, Japan). With 500 mM monovalent ion salt, the AuNPs aggregated, as shown in Fig. 4e. The electron microscopic images clearly define the spherical shape of AuNPs, with and without the presence of NaCl salt solution. Figure 4f,g reveal the optical morphology of the AuNP scattered and aggregated states, respectively, under atomic force microscopy (AFM, NanoScope, Ica, Veeco, USA). The scattered AuNPs in Fig. 4f were dispersed with large spaces between the particles. With the addition of NaCl, the clusters of AuNPs observed in Fig. 4g denoted the aggregated AuNPs. The particle size distribution of AuNPs with colorimetric assays was determined using ImageJ, an image processing program (https://imagej.nih.gov/ij/docs/guide/user-guide.pdf). Figure 4h shows the normal distribution of the histogram plot of AuNPs, indicating that the mean diameter of the particles was ~ 12 nm. On the other hand, Fig. 4i shows a nonnormal histogram skewed to the right, indicating the ambiguous distribution of the AuNPs. This affirms the distribution of aggregated AuNPs in the presence of positively charged monovalent ion salts. The above characterization validates the basic concept of the colorimetric assay and justifies its application with nucleic acid strands.

#### npcRNA validation with gold aggregation

**Figure 5:**
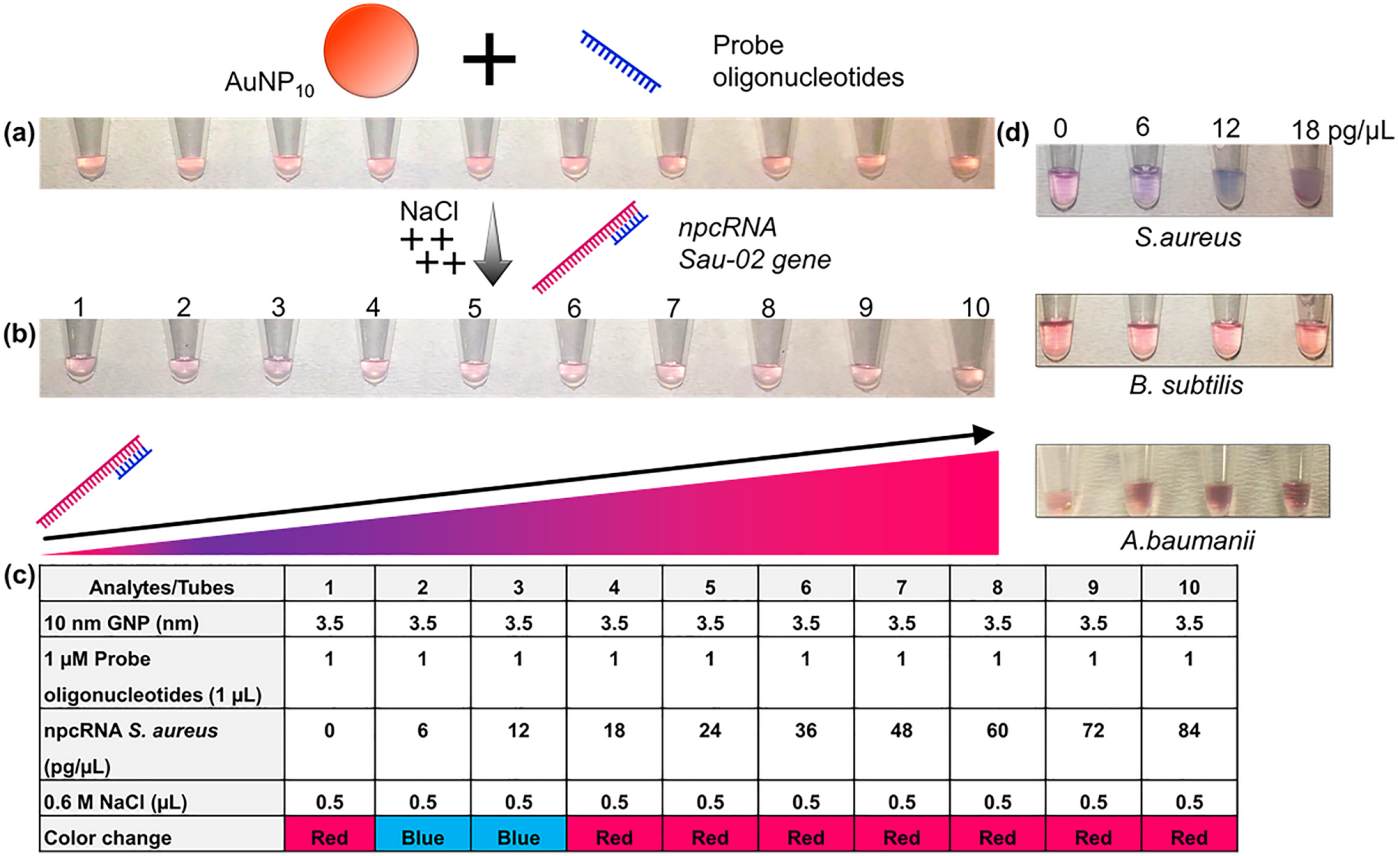
Detection of the npcRNA gene of *S. aureus* with a colorimetric assay. (**a**) Tubes and their color, containing AuNPs and probe oligonucleotides. (**b**) Tubes with changes in color after the addition of target npcRNA and NaCl salt solution. (**c**) Table showing the analyte, concentration and end color observed in all tubes. (**d**) specificity analysis. Three different pathogenic bacterial species are compared.

The hybridization between the npcRNA gene sequence and the designed probe oligonucleotide specifies the recognition of pathogenic *S. aureus* bacteria. Recognition was examined through a colorimetric assay, as shown in Fig. 5. The colorimetric assay was investigated as the probe oligonucleotide, and 10 nm AuNPs were added into ten tubes, as shown in Fig. 5a. Since the sensitivity and specificity of the complementary probe and npcRNA are significant, the assay was performed without probe-target premixing. Hence, the npcRNA and monovalent ion NaCl solutions were added into the tubes. Figure 5b shows that a color change occurred at each of the tubes as the concentration of the npcRNA gene increased. Tube numbered 1 shows the control set, where only probe oligonucleotides were added and the color of AuNPs remained red due to the strong electrostatic interaction between the single-stranded probe oligonucleotide and AuNPs. As the npcRNA gene sequence was added, a purplish color was observed in tubes 2 and 3. This result indicates that hybridization took place between the probe and target gene, where no free-floating probes were available to interact with AuNPs. Thus, the monovalent salt solution aggregated the AuNPs, and the color of the solution turned purple. Hybridization occurred at target npcRNA with concentrations as low as 6 pg/µL (Tube 2). The color of the solution regained its red color as the concentration of target increased from tube 4 up to tube 10. This phenomenon was observed due to the excess npcRNAs available in the system, which have affinity toward AuNPs. The genes were adsorbed onto and potentially capped the surface of the AuNPs, which presumably were available for aggregation in the presence of monovalent interparticle NaCl (aq)^35^. Figure 5c shows a table summarizing the content of each reaction tube with its respective analyte concentrations along with the observed color change of AuNPs. The specificity of npcRNA gene of *S. aureus* detection with colorimetric assay was compared with other bacterial species, as shown in Fig. 5d. The specificity analysis justifies the npcRNA gene of *S. aureus* detection using AuNP based colorimetric assay.

## Conclusion

Colorimetric assays for detecting *S. aureus* npcRNA were presented in the research as the preliminary validation assay for recognizing the presence of pathogenic gram-positive bacteria. The exuberant properties of AuNPs are well utilized in the field of biodiagnostics. As per the colorimetric assay, the stability of AuNPs against the salt solution was applied as the basic concept to determine npcRNA. The degree of dispersed and aggregated states of AuNPs were optimized with the AuNP size and type of salt solutions. The results affirmed that 10 nm AuNP stability in monovalent salt solution is well suited for the npcRNA gene sequence-based colorimetric assay. Probe oligonucleotides were designed as the complementary target npcRNA sequence. The aggregation of AuNPs was investigated with the hybridization of probe-npcRNA, and the detection of *S. aureus* bacteria was determined. The accuracy of the colorimetric assay was further justified through plasmon resonance absorbance results of AuNPs at 520 nm in the plasmon spectrum. Based on the results obtained, a colorimetric assay performed with 10 nm AuNPs and monovalent ion NaCl salt solution detected the npcRNA of *S. aureus* with the lowest concentration at 6 pg/µL.

## Supplementary Information

Supplementary Information.

## Data Availability

The datasets analysed during the current study are available in the BLASTN repository [Accession number NC 002,745 (npcRNA location: 1,006,365:1,006,481)].
