# Supplementary material for: Non-protein coding RNA sequences mediate specific colorimetric detection of Staphylococcus aureus on unmodified gold nanoparticles
Source: Sci Rep. 2022 Jul 23;12:12621. doi: 10.1038/s41598-022-16551-2 (PMC9308785; doi:10.1038/s41598-022-16551-2)
Supplement: Supplementary file 1 — Supplementary Information. [file 41598_2022_16551_MOESM1_ESM.pdf]

Supplementary Information

Non-Protein Coding RNA Sequences Mediate Specific Colorimetric Detection of *Staphylococcus aureus* on Unmodified Gold Nanoparticles

Subash C. B. Gopinath[1,2,3]*, Santheraleka Ramanathan[1], Suresh V. Chinni[3,4]*, Vicneswarry Dorairaj[3], Thangavel Lakshmipriya[1]

[1]Institute of Nano Electronic Engineering, Universiti Malaysia Perlis, 01000 Kangar, Perlis, Malaysia.

[2]Faculty of Chemical Engineering Technology, Universiti Malaysia Perlis, 02600 Arau, Perlis, Malaysia.

[3]Centre of Excellence for Nanobiotechnology and Nanomedicine (CoExNano), Faculty of Applied Sciences, AIMST University, Semeling, 08100 Kedah, Malaysia.

[4]Department of Biotechnology, Faculty of Applied Sciences, AIMST University, Bedong, Malaysia

*Correspondence to: subash@unimap.edu.my/v_suresh@aimst.edu.my

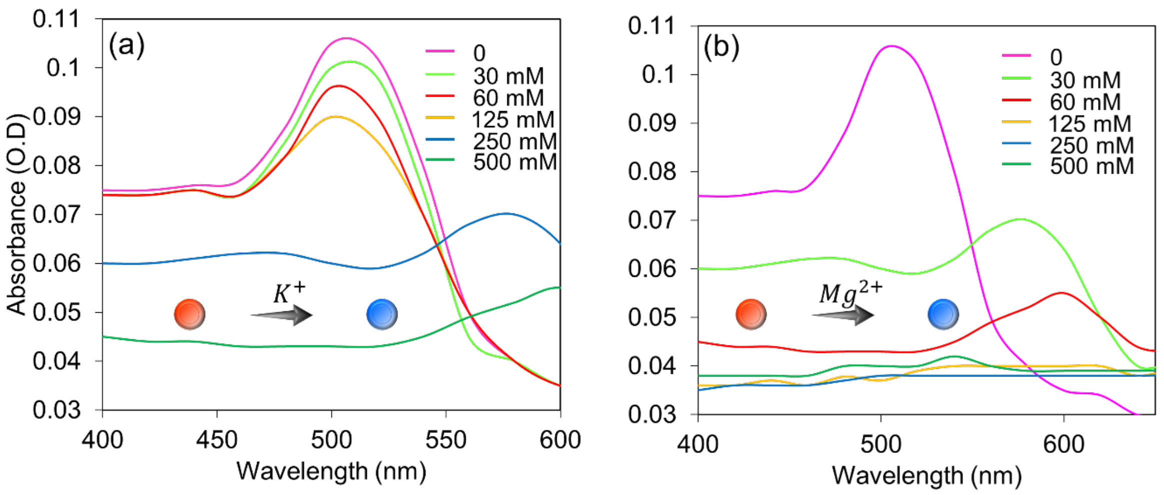

Figure S1: Graph showing the trend in the plasma spectrum, indicating the stability of 15 nm AuNPs at different (a) KCl and (b) $MgCl_2$ concentrations. The plasma spectrums show good agreement with the digital images of the colorimetric assay shown in Figure 2d and 3d.
